# Revised scored Sensory Perception Quotient reveals sensory hypersensitivity in women with autism

**DOI:** 10.1186/s13229-019-0289-x

**Published:** 2020-03-02

**Authors:** Emily Taylor, Rosemary Holt, Teresa Tavassoli, Chris Ashwin, Simon Baron-Cohen

**Affiliations:** 1 Autism Research Centre, Department of Psychiatry, University of Cambridge, Douglas House, 18b Trumpington Rd, Cambridge, CB2 8AH UK; 2 School of Psychology & Clinical Language Sciences, University of Reading, Harry Pitt Building, Earley Gate, Reading, RG6 7BE UK; 3 Centre for Applied Autism Research, Department of Psychology, University of Bath, Bath, BA2 7AY UK; 4 Cambridgeshire and Peterborough NHS Foundation Trust, CLASS Clinic, Cambridge, CB21 5EF UK

**Keywords:** Autism spectrum conditions, Sensory, Sensory Perception Quotient, Hypersensitivity, Broader autism phenotype, females

## Abstract

**Background:**

Previous research using the Sensory Perception Quotient (SPQ) has reported greater sensory hypersensitivity in people with autism spectrum condition (ASC) compared to controls, consistent with other research. However, current scoring of the SPQ does not differentiate between hyper and hyposensitivity, making it uncertain whether individuals with ASC might also show differences in hyposensitivity. Furthermore, no research to date has focused on sensory differences in females, and whether differences in sensory sensitivity extend to the broader autism phenotype (BAP). The present study aimed to fill these gaps.

**Methods:**

The present study developed and validated a Revised Scoring of the Sensory Perception Quotient (SPQ-RS) in order to investigate self-reported hypersensitivity and hyposensitivity in three groups of adults: a female ASC group (*n* = 152), mothers of children with ASC (BAP mothers group; *n* = 103), and a control mothers group (*n* = 74). All participants completed the SPQ as a self-report measure of sensory processing and the Autism-Spectrum Quotient (AQ) as a measure of the degree of autism traits.

**Results:**

The female ASC group reported significantly more hypersensitivity, but not more hyposensitivity, compared to the control female and BAP mothers groups. The BAP mothers group did not differ from the control mothers group in either reported hypersensitivity (*p* = .365) or hyposensitivity (*p* = .075), suggesting atypical sensory sensitivity is not a BAP trait within females. SPQ-RS hypersensitivity scores positively correlated with autistic traits in the female ASC (*r* = .266) and BAP mothers groups (*r* = .350).

**Conclusions:**

The present findings revealed greater sensory hypersensitivity, but not hyposensitivity, in females with ASC compared to BAP and control female groups, and that a greater degree of autism traits relates to higher hypersensitivity in ASC females. The results offer support for the enhanced perceptual functioning model using large samples of females, who are an understudied population, and demonstrate the validity of the SPQ-RS as a valuable new research tool for exploring self-reported hypersensitivity and hyposensitivity.

## Background

Autism spectrum conditions (ASC) are neurodevelopmental conditions characterised by life-long difficulties in social communication and interaction, difficulties adjusting to unexpected change, alongside unusually narrow restricted, repetitive patterns of behaviours or interests, and sensory hypersensitivity [1]. ASC has an estimated prevalence of 1% [2]. The term ‘Autism Spectrum Condition’ is favoured over ‘Autism Spectrum Disorder’, following proposals that ASC is less stigmatising and captures both the disabilities and the strengths associated with the diagnosis [3]. The broader autism phenotype (BAP) is a term describing subclinical-level traits that are qualitatively similar to characteristics of ASC, such as communication difficulties and rigidity of behaviours [4], but are observed in the general population [5]. Relatives of individuals with ASC are consistently reported to exhibit the BAP, arguably due to a shared genetic vulnerability with the ASC phenotype [6].

Following extensive research reporting that atypical sensory processing is a specific, universal and unique symptom of ASC [7], atypical sensory processing is now included within the latest diagnostic criterion of ASC [1]. Sensory processing involves the effective reception, organisation, integration, and interpretation of bodily and environmental sensory input [8]. Key indicators of atypical sensory processing as described in the DSM-5 are behaviours that are either over-responsive (hyper-reactivity) or under-responsive (hypo-reactivity) to sensory input [9]. Atypical sensory reactivity has been observed since the earliest accounts of ASC, as Kanner [10] reported that children with ASC were often fearful of loud noises and sensations resulting from the movement of objects. Moreover, Asperger [11] identified that reactivity to sensations was highly context dependent, with individuals appearing hyper-reactive to noise in one scenario, but hypo-reactive in another. Subsequently, atypical sensory reactivity has been consistently observed in individuals with ASC and is an early indicator of the condition, with reliable identification in children as young as 6 months old [12]. First-hand anecdotal reports of ASC have further elucidated the extent of atypical sensory processing, describing how the type of reactivity to sensory input varies considerably within the same individual [9]. For example, individuals have reported hyper-reactivity to light and texture but, in contrast, hypo-reactivity to pain and the calling of their name [9].

Questionnaire measures have frequently been used to investigate sensory reactivity in ASC as they are an accessible, quick, and easy tool for collecting data in larger samples. The parent-report Sensory Profile (SP) [13] and the self-report Adolescent/Adult Sensory Profile (AASP) [14] are the most widely used questionnaires, and studies using these measures have consistently shown sensory reactivity differences in ASC compared to controls across sensory modalities in approximately 90% of children and adults with ASC, irrespective of intellectual disabilities and comorbidities [15–19]. AASP responses are classified into four quadrants based on detection thresholds to stimuli, defined as sensory sensitivity (high vs. low), and behavioural responses (active vs. passive), namely: ‘low registration’ (high threshold, passive response), ‘sensation seeking’ (high threshold, active response), ‘sensory sensitivity’ (low threshold, passive response) and ‘sensation avoiding’ (low threshold, active response). The majority of individuals with ASC differ from controls in three or four quadrants, suggesting they experience contradictory reactivity [16, 19]. For example, Crane et al. [16] found 78% of their autistic sample reported sensory avoiding behaviours (hypo-reactivity); however, 44% of the same sample also reported sensory-seeking (hyper-reactivity) behaviours. Such findings of variable and often contradictory sensory reactivity have been corroborated using other questionnaire measures [20, 21], with reactivity profiles found to be highly dependent on sensory modality. For instance, Tavassoli et al. [20] identified 33% of their autistic sample to report tactical hyper-reactivity, but in contrast, only 4.4% reported visual hyper-reactivity with rather 33% reporting visual sensory-seeking behaviours. Together with the observational and anecdotal reports, these findings provide considerable evidence that individuals with ASC differ in their reactivity to sensory stimuli compared to controls and that the same individual can exhibit behaviour that appears to represent variable sensory responses.

The AASP has also been used to explore sensory reactivity in the BAP. As relatives of individuals with ASC commonly exhibit the BAP, they are a frequently studied population when aiming to identify potential BAP traits [6]. Uljarevic et al. [22] reported that 98% of mothers of children with ASC scored one or more standard deviations above normative means and 44% of them scored two or more standard deviations above normative means in at least one AASP quadrant. Thus, these mothers demonstrated atypical sensory reactivity similar to individuals with ASC, but to a lesser extent, suggesting atypical sensory reactivity is a BAP trait. However, this study was limited by a lack of control group and failure to consider whether mothers exhibited similar sensory reactivity to their child. Addressing these limitations, Glod et al. [23] compared AASP scores of parent (majority mothers)-child dyads for children with and without ASC. Parents of children with ASC differed from controls on three AASP quadrants, with significant agreement within dyads for quadrant scores (e.g. both parent and child high in sensory avoiding). This supports atypical sensory reactivity as a BAP trait.

### Evidence of atypical sensory sensitivity

Whilst atypical sensory reactivity in ASC is well documented in previous research, the underlying mechanism resulting in these atypicalities remains unclear. One fundamental determination required is to establish if atypical sensory reactivity results from atypical sensitivity to sensory stimuli, defined here as the low-level detection of sensory input, or rather atypical higher-order affective and behavioural responses to the sensations detected. To address this, Tavassoli et al. [24] developed the Sensory Perception Quotient (SPQ), a 92-item self-report questionnaire assessing basic sensory sensitivity only, rather than subsequent reactivity towards stimuli, across modalities. To develop the SPQ, Tavassoli et al. [24] adapted the well-established and validated AASP, such that items assessed primarily sensory sensitivity, with affective and behavioural responses removed. For example, the AASP item ‘I avoid escalators and/or elevators because I dislike the movement’ was adapted to ‘I would be able to tell when an elevator/lift started moving’. The SPQ has been reported in one study to date, where adults with ASC were found to be more hypersensitivity to stimuli compared to controls across all sensory modalities, except smell [24]. Sex differences within the ASC group were identified, with females scoring higher for hypersensitivity compared to males. Furthermore, SPQ and AQ scores correlated in the ASC sample and marginally in the control sample, suggesting greater hypersensitivity is associated with more autistic traits.

The SPQ is considered a reliable and valid measure of sensory sensitivity, with high split-half reliability and excellent internal consistency [24]. As a novel measure, limited comparisons between the SPQ and existing measures have been conducted. However, Tavassoli et al. demonstrated concurrent validity of the SPQ, through assessing the association of the SPQ with a previously validated measure: the Sensory Over-responsivity Scale (SensOR). The SensOR is a questionnaire measure assessing sensory over-responsivity through items asking how many sensations are experienced as aversive. SPQ and SensOR items demonstrated moderate correlations within and across groups.

In the original study by Tavassoli et al. [﻿24﻿], the SPQ was scored using a single dimensional scale which ranged from 0 (indicating hypersensitivity) to 276 (indicating hyposensitivity), rather than having separate independent scores for hyper and hyposensitivity. Individuals were rated either as more hypersensitive or hyposensitive along this scale relative to controls, and so according to this scale, individuals could not demonstrate both dimensions of hypersensitivity and hyposensitivity. Therefore, the result from the study by Tavassoli et al. [24] indicated that individuals with ASC were more hypersensitive than hyposensitive, not that individuals exclusively report hypersensitivity per se. Since the original scoring of the SPQ was not able to differentiate hypersensitivity independently from hyposensitivity, a definitive conclusion that individuals with ASC do not demonstrate hyposensitivity using a self-report measure was not possible.

The present study aims to develop and validate a Revised Scoring of the Sensory Perception Quotient (SPQ-RS), a new scoring system for the self-report SPQ to create a tool for delineating measures of both hypersensitivity and hyposensitivity, in general and for each individual sensory modality. Since no previous studies have reported about sensory sensitivity in the BAP, the present study also aims to test if the atypical sensory reactivity previously identified in the BAP [22, 23] results from atypical sensory sensitivity. Although sex differences in the presentation of autistic traits are commonly found throughout ASC research [25] and females experience more lifetime difficulties as a result of atypical sensory processing compared to males [26], males have been disproportionately represented in previous studies of sensory processing in ASC. This has resulted in poor understanding of sex differences and the sensory processing of females. Therefore, the current study will use the SPQ-RS to test sensory differences in female adults with ASC (female ASC group), mothers of children with ASC (BAP mothers group) and a female mother control sample (control mothers group). A final aim is to test the relationship between sensory sensitivity scores and degree of autistic traits, as measured by AQ, within all groups in the study.

Following the findings of Tavassoli et al. [24], we predicted that ASC females will score higher than control mothers on the SPQ-RS hypersensitivity and hyposensitivity scales. This would reflect greater differences in sensory sensitivity in ASC in general, including both hypersensitivity and hyposensitivity domains. We also predicted that these findings would be evident across all sensory modality subdomains, showing that atypical sensory sensitivity is independent of sensory modality in ASC. Furthermore, following Uljarevic et al.’s [22] and Glod et al.’s [23] findings, BAP mothers were expected to score higher than control mothers, but lower than ASC females on the SPQ-RS scales, suggesting subclinical levels of atypical sensory sensitivity, and thus that atypical sensory sensitivity is a BAP trait. Finally, it was hypothesised that AQ and SPQ-RS scale scores will positively correlate with each other in all the groups, indicating more atypical sensory sensitivity is associated with more autistic traits.

## Method

### Participants

There were 329 females recruited in total for the study, with each participant assigned to one of three different groups including: (1) adults with ASC (female ASC group), (2) mothers of a child diagnosed with ASC (BAP mothers group), and (3) a control mothers group (see Table 1). All participants were recruited online via an email notification sent to individuals registered to two University of Cambridge databases. The Autism Research Centre database (accessible at www.autismresearchcentre.com) was used to recruit adult females with ASC and BAP mothers, while a second database (accessible at www.cambridgepsychology.com) was used to recruit the control mothers sample. Participants received no incentives for participation.

**Table 1:** Participant characteristics

Characteristic	Group		
	Female ASC	BAP Mothers	Control Mothers
Number of participants	152	103	74
Mean age in years (*SD*)	40.7 (9.8)	42.9 (6.8)	42.8 (7.8)
Mean AQ (*SD*)	39.7 (6.5)	16.5 (8.3)	14.9 (6.6)
Diagnosis of one or more non-ASC psychiatric disorders (%)	73.7	31.7	36.5
Diagnosis of one or more affective disorders (%)	65.1	28.8	28.4
SPQ-RS Hypersensitivity score (*SD*)	35.1 (13.1)	22.1 (11.9)	25.0 (10.4)
SPQ-RS Hyposensitivity score (*SD*)	10.5 (6.0)	11.7 (4.7)	9.9 (5.3)

ASC: Autism Spectrum Condition; AQ: Autism-Spectrum Quotient; BAP: Broader Autism Phenotype; SD: Standard Deviation; SPQ-RS: Revised Scored Sensory Perception Quotient

The female ASC group (mean age = 40.7 years, standard deviation (SD) = 9.8 years; mean AQ = 39.7, SD = 6.5) comprised 152 female adults who had a diagnosis of ASC by a qualified professional using international criteria [1], established through the self-reporting of when, where and by whom their diagnosis was made. Where partial diagnostic information was provided (*N* = 28), the standard inclusion criterion of an AQ greater than 26 was used [27]. Six participants from the original sample of 158 recruited were excluded from the final sample used for analyses based on not scoring over 26 on the AQ. Of those who provided full details of diagnosis, 91.1% reported Asperger syndrome/high-functioning autism, 6.5% autism and 2.4% other. Participants in this group were not included or excluded for being a mother, but were aged-matched to the other two groups. This sample included a subgroup of 23 females with ASC who had a child, but the mean age and AQ scores for this subgroup were comparable to the non-mothers within this ASC group. Additionally, the mean AQ for this subgroup of ASC mothers was comparable to AQ scores reported for adult female ASC samples in a previously published extensive review (*M* = 38.8) [28], and in the present study was significantly greater than the mean AQ scores for the BAP mothers (*p* < .001) and control mothers (*p* < .001) groups.

The BAP mothers group (mean age = 42.9 years, SD = 6.8 years; mean AQ = 16.5, SD = 8.3) comprised 103 mothers without an ASC diagnosis themselves, but who reported that they had at least one child diagnosed with ASC by a qualified professional according to international criteria [1]. This was established through the self-reporting of when, where and by whom their child’s diagnosis was made. Since these participants have a relative with a diagnosis of ASC they are considered to more likely exhibit the BAP. The mean AQ of this group was comparable to samples of mothers of children with ASC (*M* = 16.4) in previous studies [29]. Thirty-two participants who suspected they may have ASC were excluded from the total sample who were initially recruited, leaving the total of 103 that were included in the data for analyses.

The control mothers group (mean age = 42.8 years, SD = 7.8 years; mean AQ = 14.9, SD = 6.6) comprised 74 mothers without an ASC diagnosis themselves and who had no child with an ASC diagnosis. The mean AQ of this group was slightly higher than previous non-ASC female samples (*M* = 12.73) [28] and did not significantly differ from the BAP mothers group (*p* = .400), indicating this group had more autistic traits than the general population. Twenty-five participants who suspected they had ASC or who scored above 26 on the AQ were excluded from the total sample of 99 who were initially recruited, leaving the total of 74 that were included in the data for analyses.

Due to the high prevalence of non-ASC psychiatric disorders in ASC and BAP populations [30], participants with a history of a psychiatric diagnosis other than ASC were not excluded from the study. Participants were asked to self-report any psychiatric disorders diagnosed by a clinician. Affective disorders (depression, bipolar disorder and anxiety disorder) were the conditions most commonly reported by participants (82.4% of people reporting non-ASC psychiatric conditions, reported at least one affective disorder). Other reported conditions included dyspraxia, anorexia nervosa, and ADHD. Diagnosis of one or more non-ASC psychiatric conditions was highly prevalent in the female ASC group (73.7% of the sample), but less so in the BAP mothers (31.7%) and control mothers (36.5%) groups. The three groups did not significantly differ from each other in age, *F*(2,328) = 2.581, *p* = .077.

### Measures

#### *Autism-Spectrum Quotient*

The adult Autism-Spectrum Quotient (AQ) is a 50-item self-report questionnaire measuring the degree of autistic traits [31]. AQ items explore traits relating to core ASC symptoms, including social skills, communication, attention switching, attention to detail and imagination. Individuals respond to item statements such as ‘I find social situations easy’, using a four-point Likert scale: ‘definitely agree’, ‘slightly agree’, ‘slightly disagree’ and ‘definitely disagree’. Responses of agreement, definitely or slightly, and disagreement, definitely or slightly, are assigned scores of 1 and 0, respectively. Half of the items are reverse scored to reduce response bias. Total scores range from 0 to 50, with higher scores indicating more autistic traits. The AQ has demonstrated good test-retest reliability [32], cross-cultural replicability [33], and high sensitivity and specificity in clinical and non-clinical samples [28].

#### *Sensory Perception Quotient*

The Sensory Perception Quotient (SPQ) is a 92-item adult self-report questionnaire measuring sensory sensitivity across modalities, using a multi-dimensional scale from hypersensitivity to hyposensitivity [24]. Individuals respond to item statements such as ‘I would be able to tell when an elevator/lift started moving’, using a four-point Likert scale, scored: 0 = ‘strongly disagree’, 1 = ‘disagree’, 2 = ‘agree’ and 3 = ‘strongly agree’. Half of the items are reverse scored to reduce response bias. Total scores range from 0 to 276, with lower scores indicating more hypersensitivity to stimuli and higher scores indicating more hyposensitivity. The SPQ also produces subscales scores for five individual sensory modalities (touch, hearing, vision, smell and taste). Tavassoli et al. [24] reported mean SPQ scores of 109 (SD = 20) for control and 93 (SD = 27) for ASC samples.

#### Revised Scoring of the Sensory Perception Quotient (SPQ-RS)

The SPQ scoring method was revised in the present study, in order to obtain scores on two SPQ-RS scales: hypersensitivity and hyposensitivity. Total scores on these scales can be individually broken down to produce five sensory modality subdomains for each scale (e.g. hypersensitivity touch, hypersensitivity hearing etc.). Responses using the SPQ-RS were coded such that higher scores on both total and subdomain scores of the SPQ-RS indicated more atypical sensory sensitivity: 0 = ‘strongly disagree’ and ‘disagree’, 1 = ‘agree’ and 2 = ‘strongly agree’. Each SPQ item was evaluated to determine whether agreeing or disagreeing with that statement indicated an atypical response, and thus should be reverse scored. For example, for the item ‘I would be able to tell when an elevator/lift started moving’, the typical response would be agreement with the statement. Therefore, disagreeing would indicate atypical sensory sensitivity and indicate hyposensitivity, and thus, this item was allocated to the hyposensitivity scale and reverse scored. This was done for each item to make it possible to independently index the degree of hyposensitivity and hypersensitivity.

Two raters evaluated each SPQ item following these steps, and there was an inter-rater agreement of 93.3% for judging and scoring the SPQ items in this way. Thirteen items identified as poor measures of sensory sensitivity were excluded from the SPQ-RS, due to textual ambiguity which those with ASC may have struggled to interpret, or indirect associations with sensory sensitivity (e.g. ‘I choose to wear muted colours’). Therefore, the SPQ-RS uses 79 of the SPQ items, with 34 items corresponding to the hypersensitivity scale (5 touch, 8 hearing, 10 vision, 6 smell and 5 taste) and 45 items corresponding to the hyposensitivity scale (12 touch, 7 hearing, 7 vision, 9 smell and 10 taste). Utilising this method, scores on the hypersensitivity scale range from 0 to 68 and on the hyposensitivity scale from 0 to 90, with a higher score indicating more atypical sensory sensitivity of the given type. Each SPQ-RS scale score can subsequently be analysed at the sensory modality subdomain level.

### Procedure

Ethical approval for the study was received from the Cambridge University Psychology Research Ethics Committee and the University of Bath Psychology Ethics Committee. Prior to recruitment, participants registered for the University of Cambridge Autism Research Centre and Psychology and Psychiatry Department databases detailed above, consenting electronically to participation and use of their data. Participants completed demographic questions, including date of birth, sex, psychiatric history and information regarding personal or familial ASC diagnoses, and could opt to receive email notifications regarding studies for which they could participate. Individuals who opted to receive these emails were invited to participate in the study.

Following recruitment, participants completed the AQ followed by the SPQ online using their own computers. All items were presented on one webpage for each questionnaire, using a multiple-choice grid with item statements listed vertically and Likert-scale responses horizontally. Participants could give only one response per item and completion of all items was required before the questionnaire was completed and submitted. There was no time limit for participants to make responses, and they could log in and out of the database as needed and save their progress on the questionnaires. Following data collection, responses were coded using the SPQ-RS.

## Results

### *Homogeneity in the****f****emale ASC group*

To test if the female ASC group was homogeneous since it contained subgroups of mothers and non-mothers, the SPQ-RS scores of mothers (*N* = 23) and non-mothers (*N* = 129) within the group were compared. Mann-Whitney *U* tests showed hypersensitivity scale scores did not differ significantly between mothers (*M* = 38.0, SD = 13.9) and non-mothers (*M* = 34.6, SD = 12.8), *U* = 1306.0, *p* = .361. Hyposensitivity scale scores also did not significantly differ between mothers (*M* = 10.8, SD = 7.0) and non-mothers (*M* = 10.5, SD = 5.8), *U* = 1472.5, *p* = .955.

Due to the high rates of self-reported comorbid psychiatric disorders in the ASC group (74%), SPQ-RS scale scores of those who with (*N* = 122) and without (*N* = 30) non-ASC diagnoses were compared. Mann-Whitney *U* tests showed hypersensitivity scale scores did not differ significantly between those with (*M* = 35.8, SD = 13.1) and without (*M* = 32.4, SD = 12.6) non-ASC diagnoses, *U* = 1616.0, *p* = .322. Hyposensitivity scale scores also did not significantly differ between those with (*M* = 10.4, SD = 6.0) and without (*M* = 10.9, SD = 6.0) non-ASC diagnoses, *U* = 1689.0, *p* = .513.

Thus, these subgroups of people within the female ASC data were analysed together as one group.

### *Overall sensory sensitivity*

Shapiro-Wilk tests showed hypersensitivity scale scores were normally distributed in the female ASC (*p* = .209) and control mothers group (*p* = .083), but not in the BAP mothers group (*p* = .002), which was slightly positively skewed. Hyposensitivity scale scores were not normally distributed in all groups (*p* < .001 for all groups), but the data for all groups showed similar positive skewness and kurtosis. Multivariate analysis of covariance (MANCOVA) with SPQ-RS hypersensitivity and hyposensitivity scale total scores as dependent variables and group as fixed factor was carried out on the data, as MANCOVAs are considered robust against non-normality when (a) dependent variables between groups are homogeneous in variance, (b) samples are moderately sized (*N* > 25 for each group), (c) skewness and kurtosis are less than |2| for all groups on the dependent variable and (d) all other assumptions of MANCOVA are met [34]. Since these applied to the present data, such tests were conducted. Since the groups differed in terms of number of participants having a non-ASC psychiatric diagnosis, this factor was included as a covariate in the analyses.

**Fig. 1:**
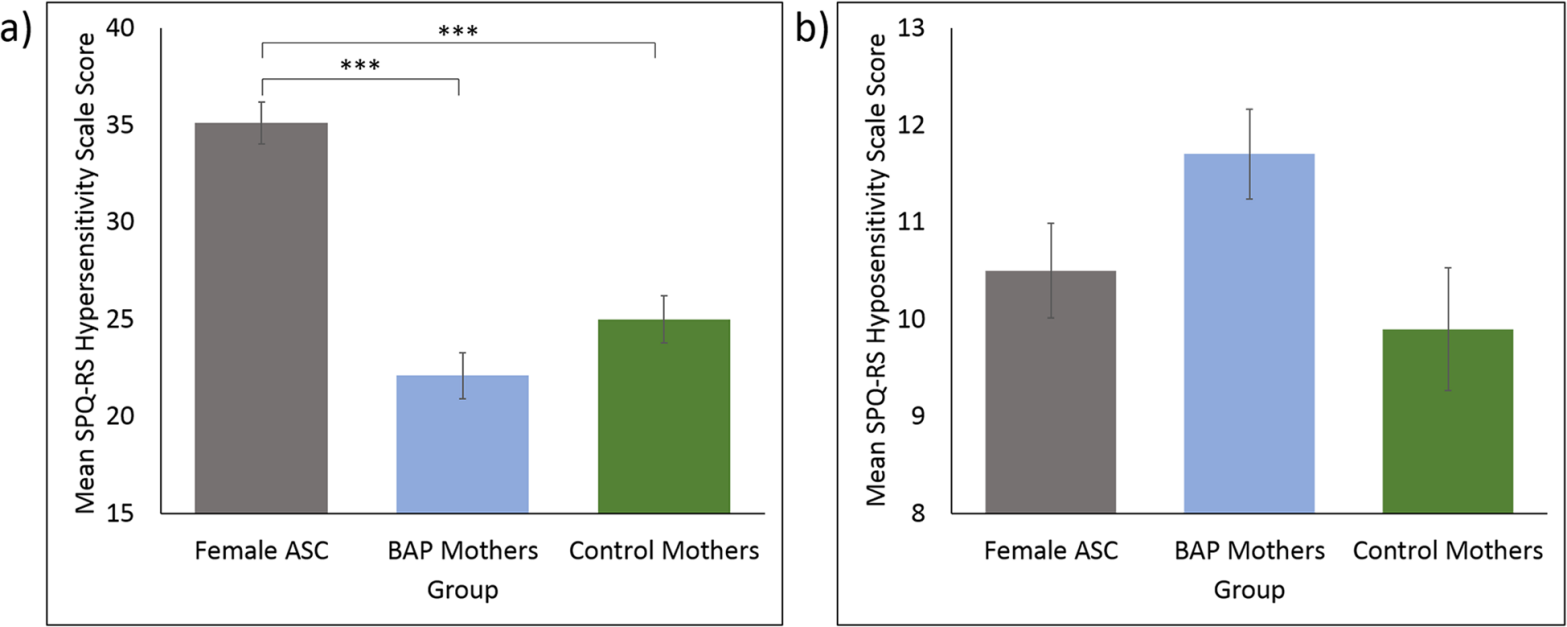
Mean **a** hypersensitivity scale (range 0–68) and **b** hyposensitivity scale (range 0–90) scores of the Revised Scored Sensory Perception Quotient (SPQ-RS). Significant pairwise comparisons are denoted, where *** indicates a significance level of *p* < .001. Error bars represent standard error of the mean. ASC, autism spectrum condition. BAP, broader autism phenotype

The results of the MANCOVA showed that the three groups differed significantly in their overall SPQ-RS scores, *F*(4, 638) = 17.413, *p* < .001; Pillai’s trace = .194; η_p_^2^ = .097. A further one-way ANCOVA showed that the groups significantly differed on the hypersensitivity scale total scores, *F*(2, 325) = 35.836, *p* < .001; η_p_^2^ = .181 (see Fig. 1). Bonferroni pairwise comparisons showed hypersensitivity scale total scores were significantly higher in the female ASC group (*M* = 35.1, SD = 13.1) compared to both the BAP mothers (*M* = 22.1, SD = 11.9; *p* < .001) and control mothers groups (*M* = 25.0, SD = 10.4; *p* < .001), while the hypersensitivity scale total scores of the BAP mothers and control mothers groups did not significantly differ from each other (*p* = .365). Another ANCOVA revealed that the three groups did not differ significantly from each other on the hyposensitivity scale total scores, *F*(2, 325) = 2.610, *p* = .075; η_p_^2^ = .016 (see Fig. 1), with the female ASC group (*M* = 10.5, SD = 6.0), the BAP mothers (*M* = 11.7, SD = 4.7), and the control mothers (*M* = 9.9, SD = 5.3) all showing comparable scores to each other.

### *Hypersensitivity sensory modality subdomains*

Results using one-way ANCOVA revealed group differences for all five of the individual sensory modality subdomains scores of the SPQ-RS hypersensitivity scale (*p* < .001 for all subdomains; see Table 2).

**Table 2:** Mean sensory modality subdomain scores of the Revised Scored Sensory Perception Quotient (SPQ-RS) hypersensitivity and hyposensitivity scales

	Group Means			Group Difference Statistics		
Variable	Female ASC (*N*=152)	BAP Mothers (*N*=103)	Control Mothers (*N*=74)	*F*	*p*	η_p_^2^
Hypersensitivity						
Touch	5.5 (2.2)	3.5 (2.2)	4.2 (2.2)	24.641	<.001	.132
Hearing	7.7 (3.6)	4.4 (3.2)	4.9 (2.8)	30.944	<.001	.160
Vision	9.3 (4.1)	5.0 (3.6)	5.5 (3.0)	37.386	<.001	.187
Smell	7.3 (3.5)	5.6 (3.1)	6.2 (3.3)	9.397	<.001	.055
Taste	5.4 (2.9)	3.5 (2.6)	4.2 (2.6)	16.573	<.001	.093
Hyposensitivity						
Touch	2.3 (2.3)	3.0 (2.3)	2.3 (1.9)	2.736	.066	.017
Hearing	3.2 (1.9)	2.6 (1.5)	2.7 (1.8)	3.734	.025	.022
Vision	1.2 (1.8)	1.3 (1.4)	.9 (1.3)	1.321	.268	.008
Smell	1.6 (2.1)	1.9 (1.9)	1.6 (2.0)	1.742	.117	.011
Taste	2.3 (2.0)	2.9 (1.8)	2.5 (1.8)	2.905	.056	.018

*Note.* Group differences between means (*F*) and respective significance levels (*p*) and effect sizes (η_p_^2^) are reported. Standard deviations are in brackets. Where Bonferroni correction was applied, significance values have been adjusted so *p* <.05 indicates statistical significance. ASC: Autism Spectrum Condition; BAP: Broader Autism Phenotype; *N*: Number of participants

**Fig. 2:**
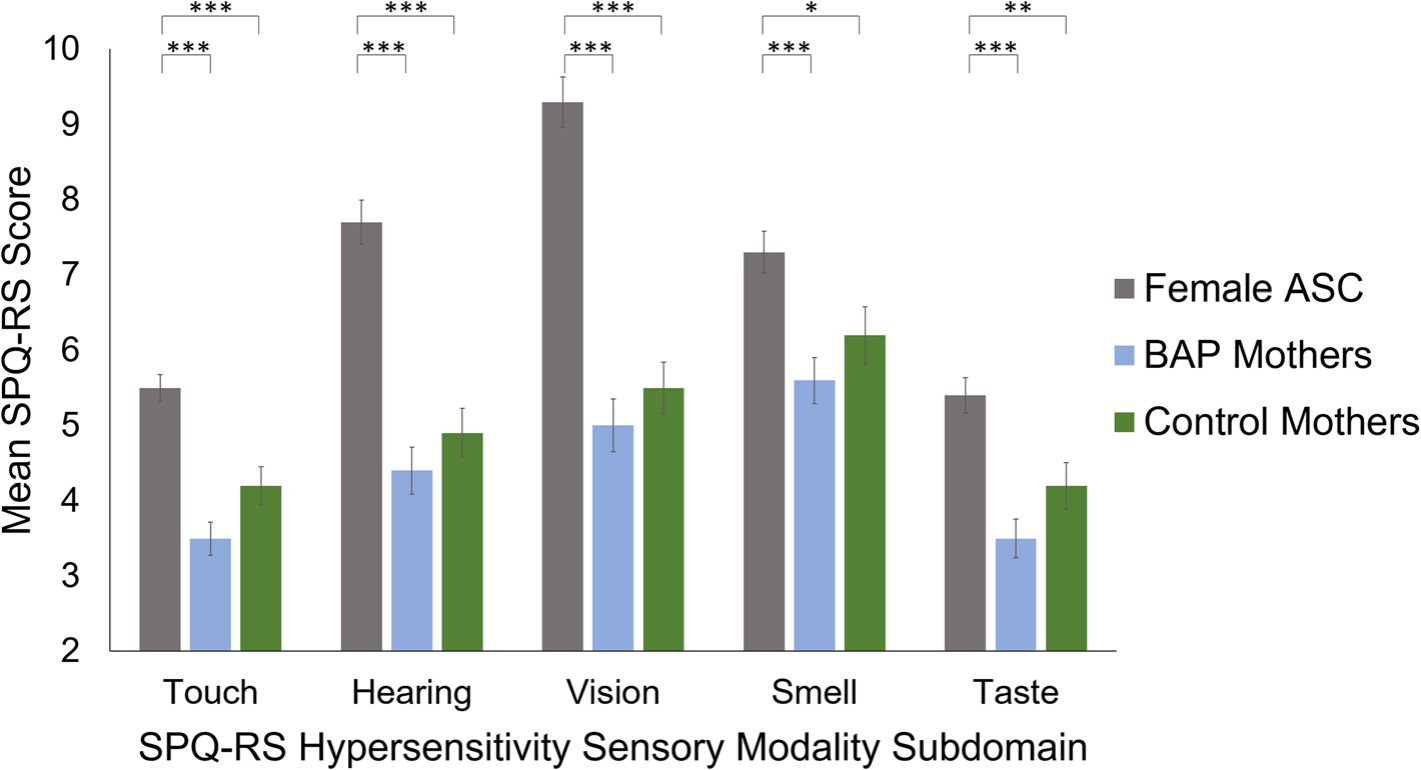
Mean hypersensitivity sensory modality subdomain scores of the Revised Scored Sensory Perception Quotient (SPQ-RS). Subdomain scores differed in range: touch = 0–10, hearing = 0–16, vision = 0–20, smell = 0–16, and taste = 0–10. Significant pairwise comparisons are denoted, where *, ** and *** indicate significance levels of *p* < .05, *p* < .01 and *p* < .001, respectively. Error bars represent standard error of the mean. ASC, autism spectrum condition. BAP, broader autism phenotype

Bonferroni pairwise comparisons showed the female ASC group scored significantly higher than both the BAP mothers (*p* < .001 for all subdomains) and the control mothers groups on all sensory modalities subdomains (touch *p* < .001, hearing *p* < .001, vision *p* < .001, smell *p* = .011, taste *p* = .003). The BAP mothers and control mothers groups did not significantly differ from each other on any of the sensory modality subdomains (touch *p* = .119, hearing *p* = 1.000, vision *p* = 1.000, smell *p* = 1.000, taste *p* = .182) (see Fig. 2).

### *Hyposensitivity sensory modality subdomains*

**Fig. 3:**
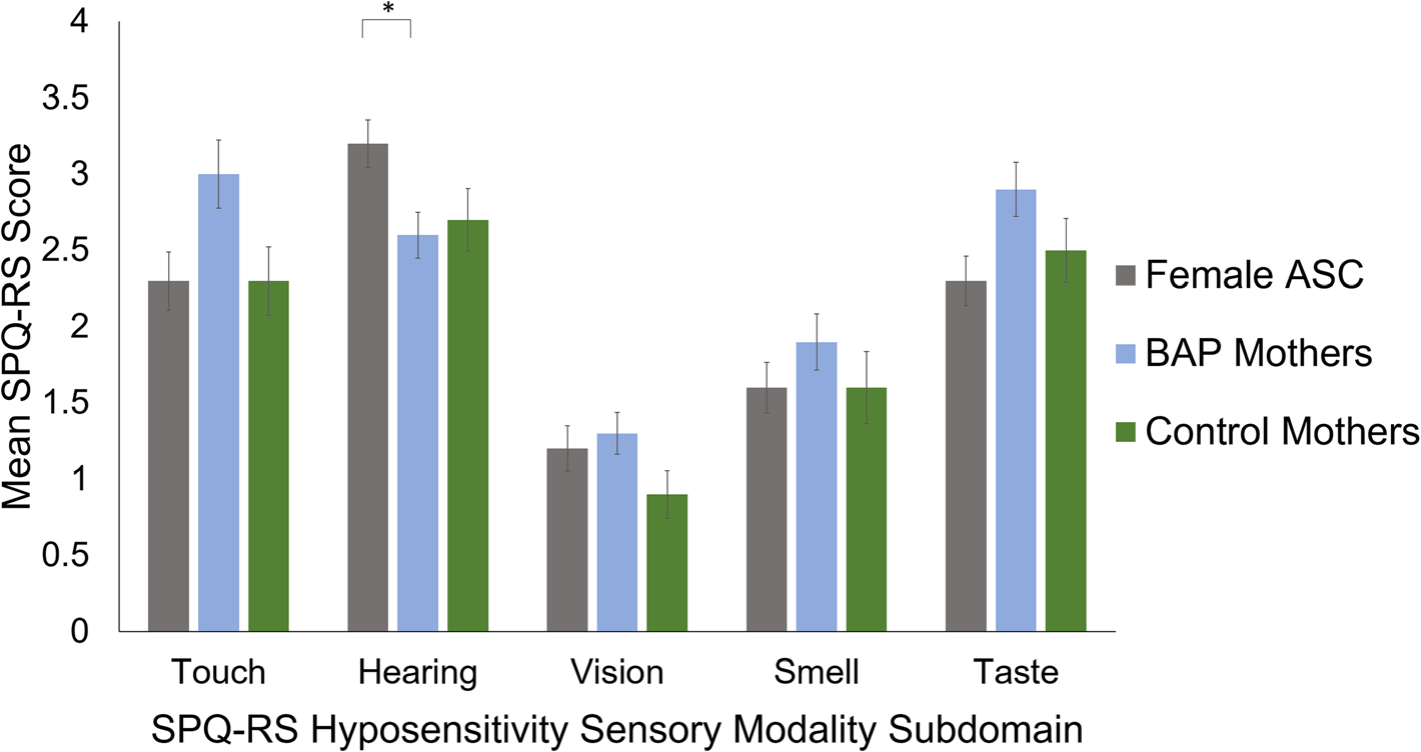
Mean hyposensitivity sensory modality subdomain scores of the Revised Scored Sensory Perception Quotient (SPQ-RS). Subdomain scores differed in range: touch = 0–24, hearing = 0–14, vision = 0–14, smell = 0–18, and taste = 0–20. Significant pairwise comparisons are denoted, where * indicates a significance level *p* < .05. Error bars represent standard error of the mean. ASC, autism spectrum condition. BAP, broader autism phenotype

Results using a one-way ANCOVA showed groups differed only on the hearing sensory modality subdomains of the SPQ-RS hyposensitivity scale (*p* = .025); see Table 2. Bonferroni pairwise comparisons on scores from the hearing subdomain showed the female ASC group scored higher than the BAP mothers (*p* = .037), but no different to the control mothers group (*p* = .123). The BAP mothers and control mothers groups did not differ significantly (*p* = 1.000) (see Fig. 3).

### *Sensory sensitivity and AQ*

**Fig. 4:**
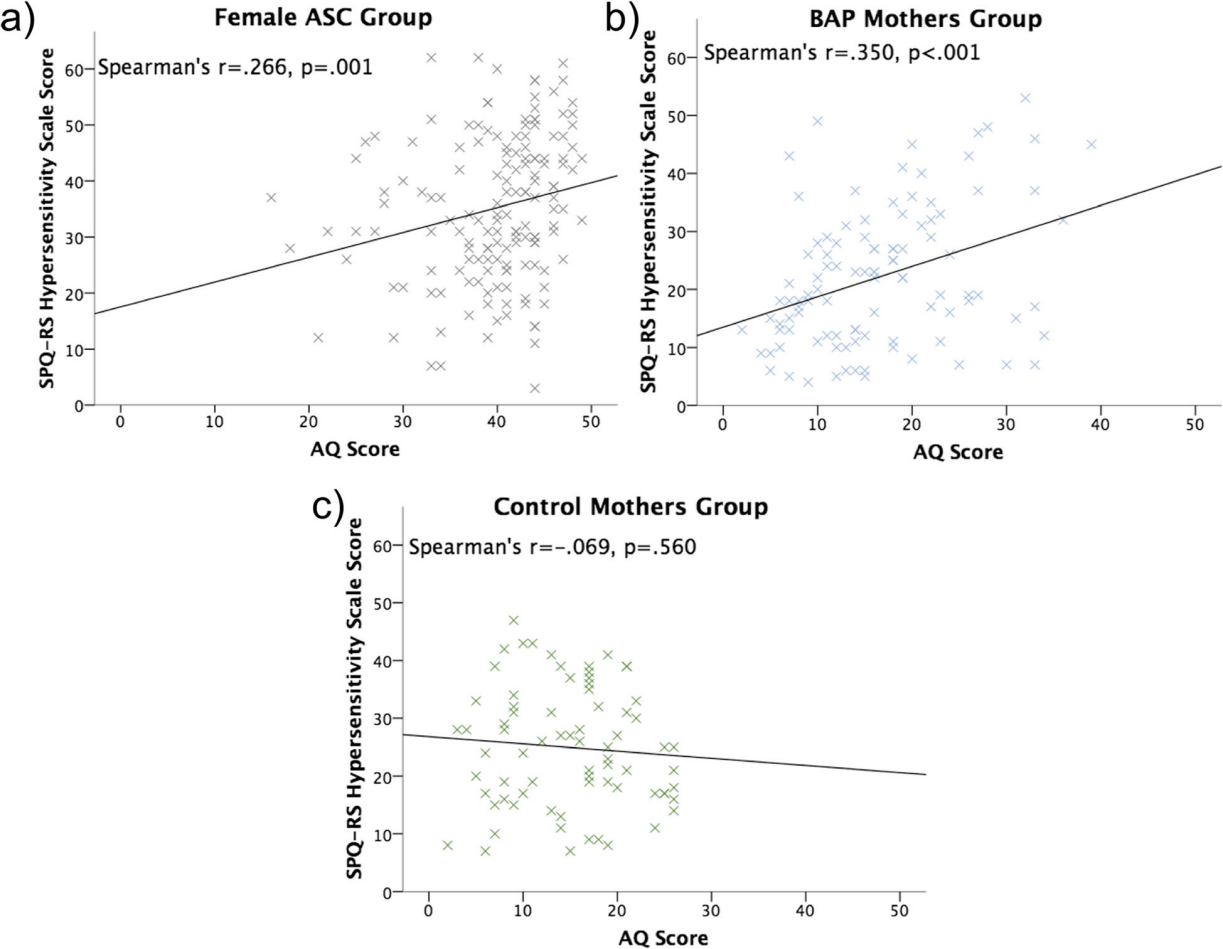
Correlation between Autism-Spectrum Quotient (AQ) and Revised Scored Sensory Perception Quotient hypersensitivity scale scores for **a** female ASC, **b** BAP mothers and **c** control mothers groups. Whilst Spearman’s rho (*r*) correlations and significance (*p*) are reported, a line of best fit based on Pearson’s correlation is presented for illustrative purposes. ASC, autism spectrum condition. BAP, broader autism phenotype

Spearman’s rho correlations showed SPQ-RS hypersensitivity scale scores moderately positively correlated with AQ scores within the female ASC (*r*_s_ = .266, *p* = .001) and BAP mothers groups (*r*_s_ = .350, *p* < .001), but not the control mothers group *(r*_s_ = − .069, *p* = .560) (Fig. 4).

**Fig. 5:**
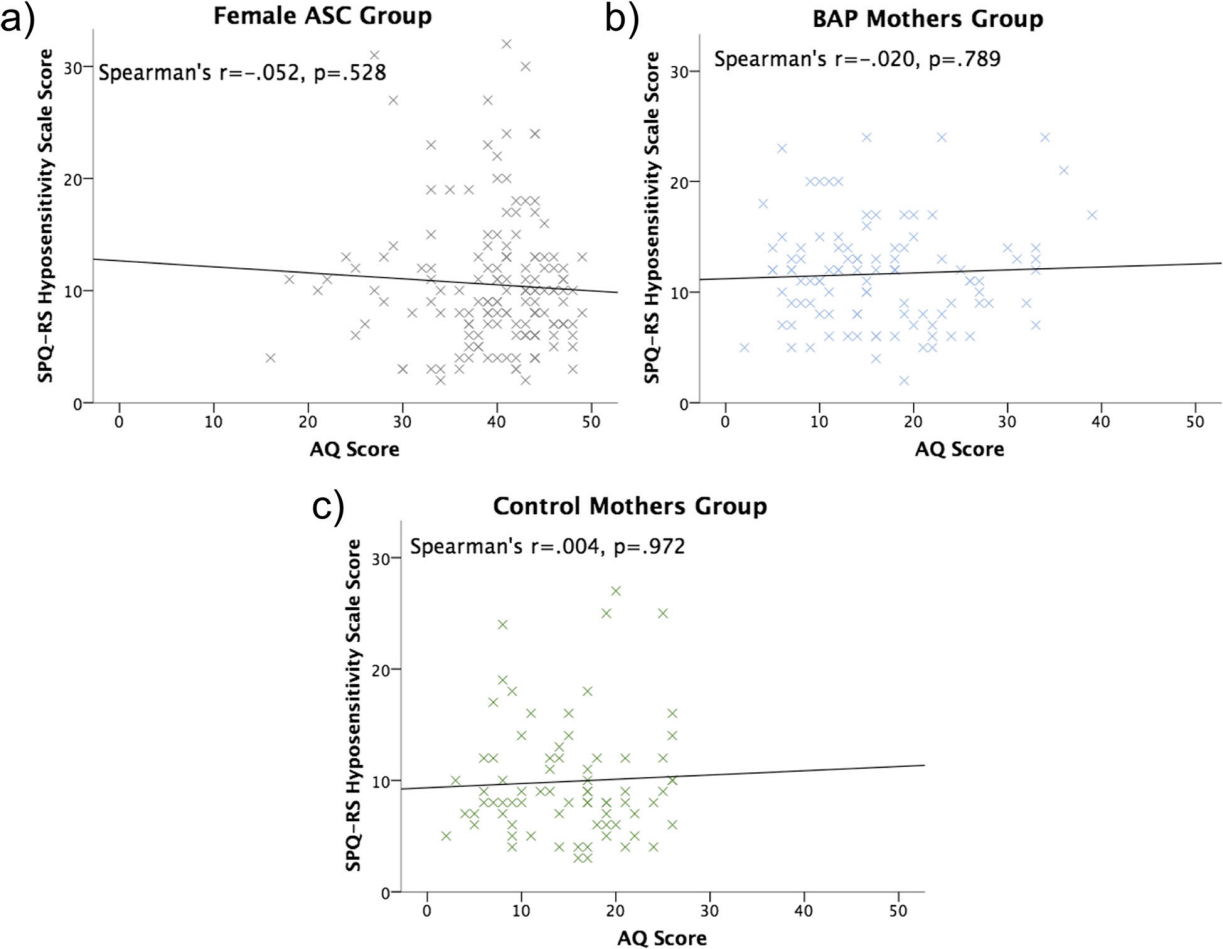
Correlation between Autism-Spectrum Quotient (AQ) and Revised Scored Sensory Perception Quotient hyposensitivity scale scores for **a** female ASC, **b** BAP mothers and **c** control mothers groups. Whilst Spearman’s rho (*r*) correlations and significance (*p*) are reported, a line of best fit based on Pearson’s correlation is presented for illustrative purposes. ASC, autism spectrum condition. BAP, broader autism phenotype

SPQ-RS hyposensitivity scale scores did not correlate with AQ scores within the female ASC (*r*_s_ = − .052, *p* = .528), BAP mothers (*r*_s_ = − .020, *p* = .789) or control mothers (*r*_s_ = .004, *p* = .972) groups (see Fig. 5).

### *Sensory sensitivity and age*

Spearman’s rho correlations within each group showed no correlation between age and SPQ-RS hypersensitivity scale scores (female ASC: *r*_s_ = .060, *p* = .463; BAP mothers: *r*_s_ = .031, *p* = .754; control mothers: *r*_s_ = − .009, *p* = .873) or age and SPQ-RS hyposensitivity scale scores (female ASC: *r*_s_ = − .050, *p* = .543; BAP mothers: *r*_s_ = − .036, *p* = .716; control mothers: *r*_s_ = − .055, *p* = .642).

## Discussion

The present results using the SPQ-RS showed that females with ASC reported more hypersensitivity to stimuli, but not hyposensitivity, compared to both control mothers and BAP mothers, consistent with other questionnaire research showing sensory hypersensitivity in those with ASC. BAP mothers and control mothers did not differ from each other in reports of hypersensitivity or hyposensitivity, suggesting atypical sensory sensitivity is not a BAP trait. Greater hypersensitivity to stimuli was associated with more autistic traits in the female ASC and BAP mothers groups, but not the control mothers group, while scores of hyposensitivity were not associated with levels of autistic traits in any sample. Together, these findings with the SPQ-RS revealed greater sensory hypersensitivity in females with ASC compared to controls, replicating previous findings with the SPQ using larger samples of females including a BAP group. These results show the SPQ-RS is a valuable and sensitive tool for measuring self-reported sensory hypersensitivity and hyposensitivity across typical and atypical groups.

This study focused on using the SPQ-RS to better characterise sensory processing in females, an understudied group within the autism spectrum. The present finding that females with ASC reported more atypical sensory sensitivity is consistent with the large clinical, questionnaire and experimental evidence base that ASC involves atypical sensory processing. The present study furthers understanding by considering differences in both hypersensitivity and hyposensitivity to stimuli. Females with ASC reported considerably more atypical sensitivity on the hypersensitivity scale of the SPQ-RS compared to female controls and mothers of children with ASC, but no differences were evident on the hyposensitivity scale. Furthermore, the female ASC group reported more hypersensitivity to the other two groups across all sensory modality subdomains, with no differences in hyposensitivity for any sensory modality subdomain. These findings show that hypersensitivity affects the sensory processing pathways of multiple modalities in females with ASC, while the lack of hyposensitivity is independent of the specific modality.

The present results further the findings reported by Tavassoli et al. [24] that males and females with ASC differ in their sensory sensitivity by reporting more hypersensitivity to stimuli on the SPQ across multiple modalities. Whilst it was hypothesised here that females with ASC would report differences in both hypersensitivity and hyposensitivity, the results only showed differences in reported hypersensitivity in individuals with ASC compared to control groups. These results are consistent with a large number of studies using experimental and physiologically based methods reporting that atypical sensory sensitivity in ASC is characterised by hypersensitivity to stimuli, rather than hyposensitivity [35–37]. Furthermore, the present study extends previous research conducted with predominantly male samples, to characterise atypical sensory sensitivity in large sample of females with ASC, indicating that atypical sensory processing is a universal feature of ASC. These findings are also aligned with the enhanced perceptual function (EPF) model of ASC, which suggests atypical sensory reactivity in ASC results from an underlying enhanced low-level sensory processing, with hypersensitivity to stimuli [38, 39].

The finding that females with ASC exclusively report hypersensitivity, with no hyposensitivity, to stimuli further supports the EPF model. Previously, the EPF model has been criticised for its inability to accommodate hypo-reactivity resulting from an underlying hyposensitivity to stimuli [40]. However, following the lack of hyposensitivity, it may suggest that hypo-reactivity identified in previous literature [16] and described in the DSM-5 diagnostic criteria may not stem from an underlying hyposensitivity, but rather from the higher-order social, communication and cognitive difficulties associated with ASC [41, 42]. For example, auditory hypo-reactivity may reflect social difficulties associated with responding appropriately to speech, or difficulties in shifting attention from one sensory input to another [43]. Further research exploring this argument, perhaps through varying levels of attention or social engagement during sensory stimulation, is required.

This study is the first to report about sensory sensitivity related to the BAP, with no differences found in SPQ-RS scores between BAP mothers and control mothers. These results suggest that mothers of children with ASC do not demonstrate atypical sensory sensitivity, and thus it is not a BAP trait. The present finding of no differences in sensory sensitivity in the BAP group compared to controls differs from previous studies reporting atypical sensory processing related to the BAP [22, 23]. However, those previous studies measured sensory reactivity while the current study measured sensory sensitivity, and there may be differences related to the BAP between these dimensions of sensory processing. While evidence has reported moderate genetic relationships with sensory reactivity [44], less is known about the genetic influence on sensory sensitivity. The finding of atypical sensory reactivity in previous research may be due to the bidirectional modelling of maladaptive behaviours: both parent and child may adopt sensory avoidant behaviours from the child modelling particular stimuli as threatening [23], rather than parent and child both experiencing hypersensitivity to the stimulus. The null finding in the present study could arguably be attributed to the subtlety of BAP traits, particularly in females. However, since no differences were found here on all sensory modality subdomains, and previous research has reported differences in the BAP related to sensory reactivity, this explanation would seem unlikely.

The present study revealed a quantitative relationship between the degree of hypersensitivity, but not hyposensitivity, and number of autistic traits. Since there were no group differences on the hyposensitivity scale, the finding of non-correlations for this scale in the various groups is unsurprising. However, hypersensitivity scale scores showed modest positive correlations with AQ scores within the ASC and BAP mothers groups, showing more severe hypersensitivity was associated with greater autistic traits in ASC and BAP groups. This is consistent with previous questionnaire-based research reporting more atypical sensory processing, including sensory sensitivity, was associated with more autistic traits [17, 24]. However, SPQ-RS hypersensitivity scale and AQ scores did not correlate within the control group, which is inconsistent with these previous reports. One possible explanation is that differences in sensory sensitivity in the general population are more subtle, and therefore may not be easily demonstrated using self-report questionnaire measures.

Within the field of developmental psychopathology, whether developmental disorders are best classified through categorical or dimensional approaches is a frequent area of debate, with significant implications for both diagnostic techniques and research practices [45]. Differences in hypersensitivity in the present study were specific to our ASC group, with no differences in hypersensitivity found for the BAP group compared to controls. Therefore, the present findings suggest that hypersensitivity is a distinct and qualitatively different characteristic of ASC, which supports a category-based approach to the measure of sensory sensitivity.

The findings show the SPQ-RS to be a valid, accessible, and useful new research tool allowing researchers and clinicians to quantify sensory sensitivities, as opposed to reactivity. In particular, the SPQ-RS is an advantageous tool to assess hypersensitivity and hyposensitivity to stimuli in general, but also by modality, for a more detailed, heuristic understanding of an adult’s sensory sensitivity. By obtaining such a detailed profile of sensory sensitivity, the SPQ-RS can be used to determine if sensitivity and modality-specific differences are associated with general atypical sensory reactivity as described in the DSM-5 criteria, but also more specific behaviours. For example, investigating whether hypersensitivity to taste is directly associated with repetitive and rigid food preferences. This may help elucidate the underlying causes of specific behavioural patterns. Further, in clinical settings, the SPQ-RS could potentially be used in combination with a measure of sensory reactivity such as the AASP, to help clarify if atypical sensory reactivity is primarily a sensory sensitivity difference or rather a maladaptive behavioural response, thus helping to inform the best approach for management of sensory symptoms.

More generally, the SPQ-RS could be used to further investigate if atypical sensory processing, specifically sensory sensitivity, contribute to the wider cognitive and behavioural patterns of ASC. Atypical sensory processing has been associated with positive aspects of ASC, including superior attention to detail and savant abilities [35], but also with ASC core symptoms outlined in the DSM-5 criteria, including maladaptive behaviours [46], increased repetitive behaviours [47] and social impairments [48]. The EPF model proposes these behaviours result directly from receiving overwhelming amounts of sensory information following hypersensitivity, with maladaptive behaviours adopted to reduce sensory input [39]. The SPQ-RS could be used in future work to determine if there are direct relationships between hypersensitivity, as identified in the present study, and these diagnostically relevant behaviours.

### Limitations

The present study has many strengths, including devising a new research tool, successfully addressing deficiencies of previous studies with important implications for theory, employing a highly-powered sample size and the use of control groups. However, some limitations should be acknowledged. Firstly, the study only included high-functioning females with ASC because participants needed to be verbal and able to self-report about their behaviour. Further, only women with a clinical diagnosis were included in the ASC group, who likely represent a relatively ‘severe’ group of females with ASC, as diagnosis of women within the sample age range is less common. Therefore, the results cannot be generalised to the wider spectrum of those with ASC, including those who are low-functioning, females with less ‘severe’ ASC, males with ASC, or those with a relative having an ASC diagnosis.

Additionally, the sample was recruited online and consequently participants were self-selecting and only those with computer and literary skills sufficient to complete questionnaires online could participate. This may have affected the nature of the samples, for example, the control females had higher levels of autistic traits compared to other control samples in previous studies. However, the present results were consistent with findings from previous research using the SPQ. Further, the control group participants were all mothers, whereas the ASC group were predominantly non-mothers, and so group differences could be attributed to motherhood. However, as no differences in sensory sensitivity were identified between mothers and non-mothers within the ASC group, we feel this is an unlikely explanation for our results. Lastly, due to the high rates of reported comorbid psychiatric disorders in the ASC group (74%), it remains unclear if the identified differences in sensory sensitivity are specific to ASC. However, the presence of non-ASC psychiatric diagnoses were included as covariates during the analyses which produced the present results, and so comorbid diagnoses were controlled for in the study. Furthermore, comparisons within the ASC group were carried out between those who reported having a comorbid diagnosis and those without comorbidities the results showed no statistically significant differences in SPQ-RS scores, between them. However, these analyses were dependent on the accurate self-reporting of psychiatric histories, and so further research is needed testing the effect of specific comorbidities on sensory sensitivity measures.

## Conclusion

The present study used a revised scoring system of the self-report SPQ (the SPQ-RS) (Additional file 1) to measure both hypersensitivity and hyposensitivity to stimuli in samples of females with and without ASC and found that females with ASC report more hypersensitivity, but not hyposensitivity, compared to control females and BAP females. There were no differences in hypersensitivity or hyposensitivity between the BAP females and control females, showing that differences in sensory sensitivity did not extend to the BAP within our female samples. Further results showed that a higher degree of autism traits was associated with greater hypersensitivity for the females with ASC and the BAP females, but not for the control females. The findings offer support for the EPF model and help validate the SPQ-RS as a new tool to better delineate sensory profiles in autism samples.

## Additional file

**Additional file 1:** Supplementary Material: The Revised Scoring of the Sensory Perception Quotient (SPQ-RS)

## Data Availability

The datasets generated and/or analysed during the current study are not publicly available as volunteers in the Cambridge Autism Research Database (CARD) did not consent for their data to be deposited in an Open Access archive. However, the CARD Management Committee considers requests by researchers for specific parts of the database (in anonymised form) to test specific hypotheses (please contact: info@autismresearchcentre.com).

## Abbreviations

AASP: Adolescent/Adult Sensory Profile
ANCOVA: Analysis of covariance
AQ: Autism-Spectrum Quotient
ASC: Autism spectrum condition
BAP: Broader autism phenotype
EPF: Enhanced perceptual functioning
MANCOVA: Multivariate analysis of covariance
SD: Standard deviation
SP: Sensory Profile
SPQ: Sensory Perception Quotient
SPQ-RS: Revised Scored Sensory Perception Quotient

## References

[1] American Psychiatric Association (2013). Diagnostic and statistical manual of mental disorders.

[2] Brugha TS, McManus S, Bankart J, Scott F, Purdon S, Smith J (2011). Epidemiology of autism spectrum disorders in adults in the community in England. Arch Gen Psychiatry.

[3] Baron-Cohen S, Scott FJ, Allison C, Williams J, Bolton P, Matthews FE (2009). Prevalence of autism-spectrum conditions: UK school-based population study. Br J Psychiatry.

[4] Sucksmith E, Roth I, Hoekstra RA (2011). Autistic traits below the clinical threshold: Re-examining the broader autism phenotype in the 21st century. Neuropsychol Rev.

[5] Bolton P, Macdonald H, Pickles A, Rios P, Goode S, Crowson M (1994). A case-control family history study of autism. J Child Psychol Psychiatry.

[6] Gerdts J, Bernier R. The broader autism phenotype and its implications on the etiology and treatment of autism spectrum disorders. Autism Res Treat. 2011:1–19. 10.1155/2011/545901. 10.1155/2011/545901 PMC3420416 22937250

[7] Bogdashina O (2013). Sensory theory in autism makes sense: a brief review of the past and present research. Open Access Autism.

[8] John AE, Mervis CB (2010). Sensory modulation impairments in children with Williams syndrome. Am J Med Genet Part C Semin Med Genet.

[9] Elwin M, Ek L, Kjellin L, Schröder A (2013). Too much or too little: hyper- and hypo-reactivity in high-functioning autism spectrum conditions. J Intellect Develop Disabil.

[10] Kanner L (1943). Autistic disturbances of affective contact. Nervous Child.

[11] Asperger H, Frith U (1991). Autistic psychopathy’ in childhood (translated by Frith U). Autism and Asperger syndrome.

[12] O’Neill M, Jones RSP (1997). Sensory-perceptual abnormalities in autism: a case for more research?. J Autism Dev Disord.

[13] Dunn W (1999). Sensory Profile.

[14] Brown C, Dunn W (2002). Adolescent-Adult Sensory Profile: user’s manual.

[15] Ben-Sasson A, Hen L, Fluss R, Cermak SA, Engel-Yeger B, Gal E (2009). A meta-analysis of sensory modulation symptoms in individuals with autism spectrum disorders. J Autism Dev Disord.

[16] Crane L, Goddard L, Pring L (2009). Sensory processing in adults with autism spectrum disorders. Autism.

[17] Horder J, Wilson CE, Mendez MA, Murphy DG (2014). Autistic traits and abnormal sensory experiences in adults. J Autism Dev Disord.

[18] Kern JK, Trivedi MH, Garver CR, Grannemann BD, Andrews AA, Savla JS (2006). The pattern of sensory processing abnormalities in autism. Autism..

[19] Kern JK, Garver CR, Carmody T, Andrews AA, Trivedi MH, Mehta JA (2007). Examining sensory quadrants in autism. Res Autism Spectr Disord.

[20] Tavassoli T, Bellesheim K, Siper PM, Wang AT, Halpern D, Gorenstein M, et al. Measuring sensory reactivity in autism spectrum disorder: application and simplification of a clinician-administered sensory observation scale. J Autism Dev Disord. 2015. 10.1007/s10803-015-2578-3 26340959

[21] Tomchek SD, Dunn W (2007). Sensory processing in children with and without autism: a comparative study using the Short Sensory Profile.

[22] Uljarević M, Prior MR, Leekam SR (2014). First evidence of sensory atypicality in mothers of children with autism spectrum disorder (ASD). Mol Autism.

[23] Glod M, Riby DM, Honey E, Rodgers J (2016). Sensory atypicalities in dyads of children with autism spectrum disorder (ASD) and their parents. Autism Res.

[24] Tavassoli T, Hoekstra RA, Baron-Cohen S (2014). The Sensory Perception Quotient (SPQ): development and validation of a new sensory questionnaire for adults with and without autism. Mol Autism.

[25] Werling DM, Geschwind DH (2013). Sex differences in autism spectrum disorders. Curr Opin Neurol.

[26] Lai M-C, Lombardo MV, Pasco G, Ruigrok ANV, Wheelwright SJ, Sadek SA (2011). A behavioral comparison of male and female adults with high functioning autism spectrum conditions. PLoS One.

[27] Woodbury-Smith MR, Robinson J, Wheelwright S, Baron-Cohen S (2005). Screening adults for Asperger syndrome using the AQ: a preliminary study of its diagnostic validity in clinical practice. J Autism Dev Disord.

[28] Ruzich E, Allison C, Smith P, Watson P, Auyeung B, Ring H (2015). Measuring autistic traits in the general population : a systematic review of the Autism-Spectrum Quotient (AQ) in a nonclinical population sample of 6, 900 typical adult males and females.

[29] Wheelwright S, Auyeung B, Allison C, Baron-Cohen S (2010). Defining the broader, medium and narrow autism phenotype among parents using the Autism Spectrum Quotient (AQ). Mol Autism.

[30] Ingersoll B, Meyer K, Becker MW (2011). Increased rates of depressed mood in mothers of children with ASD associated with the presence of the broader autism phenotype. Autism Res.

[31] Baron-Cohen S, Wheelwright S, Skinner R, Martin J, Clubley E (2001). The Autism-Spectrum Quotient (AQ): evidence from asperger syndrome/high-functioning autism, males and females, scientists and mathematicians. J Autism Dev Disord.

[32] Hoekstra RA, Bartels M, Cath DC, Boomsma DI (2008). Factor structure, reliability and criterion validity of the autism-spectrum quotient (AQ): a study in Dutch population and patient groups. J Autism Dev Disord.

[33] Wakabayashi A, Baron-Cohen S, Uchiyama T, Yoshida Y, Tojo Y, Kuroda M (2007). The Autism-Spectrum Quotient (AQ) children’s version in Japan: a cross-cultural comparison. J Autism Dev Disord.

[34] Boneau CA (1960). The effects of violations of assumptions underlying the t test. Psychol Bull.

[35] Baron-Cohen S, Ashwin E, Ashwin C, Tavassoli T, Chakrabarti B (2009). Talent in autism: hyper-systemizing, hyper-attention to detail and sensory hypersensitivity. Philos Trans R Soc Lond Ser B Biol Sci.

[36] Heaton P, Davis RE, Happé FGE (2008). Research note: exceptional absolute pitch perception for spoken words in an able adult with autism. Neuropsychologia..

[37] Ashwin E, Ashwin C, Rhydderch D, Howells J, Baron-Cohen S (2009). Eagle-eyed visual acuity: an experimental investigation of enhanced perception in autism. Biol Psychiatry.

[38] Mottron L, Dawson M, Soulières I, Hubert B, Burack J (2006). Enhanced perceptual functioning in autism: an update, and eight principles of autistic perception. J Autism Dev Disord.

[39] Mottron L, Burack JA, Dawson M, Soulières I, Hubert B. Enhanced perceptual functioning in the development of autism. Dev Autism Perspect from Theory Res. 2001;36 January 2001:27–43. doi: 10.1007/s10803-005-0040-7. 10.1007/s10803-005-0040-7 16453071

[40] Pellicano E (2013). Sensory symptoms in autism: a blooming, buzzing confusion?. Child Dev Perspect.

[41] Ashwin C, Chapman E, Howells J, Rhydderch D, Walker I, Baron-Cohen S (2014). Enhanced olfactory sensitivity in autism spectrum conditions. Mol Autism.

[42] Liss M (2006). Sensory and attention abnormalities in autistic spectrum disorders. Autism.

[43] Keehn B, Müller RA, Townsend J (2013). Atypical attentional networks and the emergence of autism. Neurosci Biobehav Rev.

[44] Goldsmith HH, Van Hulle CA, Arneson CL, Schreiber JE, Gernsbacher MA (2006). A population-based twin study of parentally reported tactile and auditory defensiveness in young children. J Abnorm Child Psychol.

[45] Coghill D, Sonuga-Barke EJS (2012). Annual research review: categories versus dimensions in the classification and conceptualisation of child and adolescent mental disorders - Implications of recent empirical study. J Child Psychol Psychiatry Allied Discip.

[46] Baker AEZ, Lane A, Angley MT, Young RL (2008). The relationship between sensory processing patterns and behavioural responsiveness in autistic disorder: a pilot study. J Autism Dev Disord.

[47] Boyd BA, McBee M, Holtzclaw T, Baranek GT, Bodfish JW (2009). Relationships among repetitive behaviors, sensory features, and executive functions in high functioning autism. Res Autism Spectr Disord.

[48] Kirby AV, Dickie VA, Baranek GT. Sensory experiences of children with autism spectrum disorder: In their own words. Autism. 2014;1–11. doi: 10.1177/1362361314520756. 10.1177/1362361314520756 PMC4556130 24519585

