# Supplementary material for: Revised scored Sensory Perception Quotient reveals sensory hypersensitivity in women with autism
Source: Mol Autism. 2020 Mar 2;11:18. doi: 10.1186/s13229-019-0289-x (PMC7053068; doi:10.1186/s13229-019-0289-x)
Supplement: Supplementary file 1 — Additional file 1: Supplementary Material: The Revised Scoring of the Sensory Perception Quotient (SPQ-RS) [file 13229_2019_289_MOESM1_ESM.docx]

## Supplementary Material: The Revised Scoring of the Sensory Perception Quotient (SPQ-RS)

For full details of the original SPQ, please see:

Tavassoli, T., Hoekstra, R. A., & Baron-Cohen, S. (2014). The Sensory Perception Quotient (SPQ): development and validation of a new sensory questionnaire for adults with and without autism. *Molecular Autism*, *5*(29), 1-10.

Participants are required to respond how strongly they agree with each statement using the following responses: ‘strongly disagree’, ‘disagree’, ‘agree’ ‘strongly agree’. Table 1 details the original Sensory Perception Quotient (SPQ) items, with the SPQ-RS scale allocation and scoring.

Table 1.

*Items of the Sensory Perception Quotient (SPQ) with the Revised Scoring of the Sensory Perception Quotient (SPQ-RS). Italicised items are reverse scored in the SPQ-RS.*

| Item No. | SPQ Item | SPQ-RS Scale | Modality |
| --- | --- | --- | --- |
| 1 | I would notice if someone added 5 grains of salt to my cup of water | Hypersensitivity | Taste |
| 2 | I would be able to distinguish different people by their smell. | Hypersensitivity | Smell |
| 3 | I wouldn’t notice if someone added a spoonful of sugar to my tea. | Hyposensitivity | Taste |
| 4 | I wouldn’t be afraid of hurting myself when falling off my bike at high speed. | Hyposensitivity | Touch |
| 5 | I wouldn’t be able to detect the motion of the blades of a rotating fan even when it is at minimum speed. | Hyposensitivity | Vision |
| 6 | The sound of a piano and a violin playing the same note seem very similar to me. | Excluded | Hearing |
| 7 | I would be able to detect if a strawberry was ripe by smell alone. | Hypersensitivity | Smell |
| 8 | I would be able to distinguish milk chocolate and dark chocolate by their taste alone. | *Hyposensitivity* | Taste |
| 9 | I cannot tolerate hot showers (above 40°C / 105°F). | Excluded | Touch |
| 10 | I wouldn’t need an anaesthetic to cope with a dental procedure, such as a cavity-filling. | Hyposensitivity | Touch |
| 11 | I would have to wait for 10 minutes for a hot drink to cool down before swallowing it, otherwise it would be too hot for me. | Hypersensitivity | Touch |
| 12 | I would be able to visually detect the change in brightness of a light each time a dimmer control is moved one notch. | *Hyposensitivity* | Vision |
| 13 | I wouldn’t be able to detect large objects, such as parked cars, clearly on a dark night. | Hyposensitivity | Vision |
| 14 | I would notice if someone added 5 drops of lemon juice to my cup of water. | *Hyposensitivity* | Taste |
| 15 | I would be the last person to detect if something was burning. | Hyposensitivity | Smell |
| 16 | I wouldn’t be able to feel the vibrations from loud music if I was sitting next to the loud speaker (e.g. at a concert). | Hyposensitivity | Touch |
| 17 | I wouldn’t be able to feel a small volume change in music as a difference in vibration on my skin. | *Hypersensitivity* | Touch |
| 18 | I can’t hear the TV when it is very quiet, even when other people can. | Hyposensitivity | Hearing |
| 19 | I would be able to hear a leaf move if blown by the wind on a quiet street. | Hypersensitivity | Hearing |
| 20 | I wouldn’t be able to taste the difference between two pieces of dark chocolate. | *Hypersensitivity* | Taste |
| 21 | I would be able to taste the difference between two brands of salty potato chips/crisps. | Excluded | Taste |
| 22 | When people are talking the words seem to merge together. | Hyposensitivity | Hearing |
| 23 | I can only look at bright colours for a brief period of time. | Hypersensitivity | Vision |
| 24 | I would lose my balance very easily if I was standing on one foot with my eyes closed. | *Hyposensitivity* | Hearing |
| 25 | I wouldn’t be able to smell a barbecue from 60 feet (20 metres) away. | Hyposensitivity | Smell |
| 26 | I can’t spin round and round without falling over. | Excluded | Hearing |
| 27 | I wouldn’t notice a 10 degree difference in temperature of the weather. | Hyposensitivity | Touch |
| 28 | I can drink tea/coffee “straight”, without needing to add milk or sugar. | Hyposensitivity | Taste |
| 29 | I can’t hear the bass in music. | Hyposensitivity | Hearing |
| 30 | I would be able to smell the difference between freshly cut grass and uncut grass. | *Hyposensitivity* | Smell |
| 31 | I wouldn’t be able to feel the label at the back of my shirt even if I thought about it. | Hyposensitivity | Touch |
| 32 | I can hear electricity humming in the walls. | Hypersensitivity | Hearing |
| 33 | I notice the flickering of a desktop computer even when it is working properly. | Hypersensitivity | Vision |
| 34 | I wouldn’t be able to tell if milk is off simply by smelling it. | Hyposensitivity | Smell |
| 35 | I would be able to notice a tiny change (e.g. 1 degree) in the temperature of the weather. | Hypersensitivity | Touch |
| 36 | I would be able to feel a one millimetre cut in my skin. | Excluded | Touch |
| 37 | I would be able to see the individual blades in a rotating fan even if it was at maximum speed. | Hypersensitivity | Vision |
| 38 | I would be able to tell the weight difference between two different coin sizes on the palm of my hand, if my eyes were closed. | *Hyposensitivity* | Touch |
| 39 | I wouldn’t get dizzy on a carousel/merry-go-round, even at high speed. | Excluded | Hearing |
| 40 | I can’t see written words on a page that other people can see. | Hyposensitivity | Vision |
| 41 | I would be able to distinguish between two oranges purely by their taste. | Hypersensitivity | Taste |
| 42 | I couldn’t distinguish a familiar person and a stranger by their smell. | Hyposensitivity | Smell |
| 43 | I couldn’t detect if bread is stale purely by its smell. | *Hypersensitivity* | Smell |
| 44 | I can’t tell if my clothes are clean or dirty by smell alone. | Hyposensitivity | Smell |
| 45 | I would be able to detect the sound of a vacuum cleaner from any room in a two storey building. | Hypersensitivity | Hearing |
| 46 | I wouldn’t notice the difference between even and uneven ground when driving over it sitting in the back seat of a car. | Hyposensitivity | Touch |
| 47 | I would be able to drink a cup of boiling water straight after it had been poured from the kettle. | Hyposensitivity | Touch |
| 48 | I couldn’t tell two types of green apples apart purely from their colour. | Excluded | Vision |
| 49 | I would be able to distinguish between an old and a new book by their smell. | *Hyposensitivity* | Smell |
| 50 | I would be able to read a street sign from a distance of 100 feet (30 metres). | Hypersensitivity | Vision |
| 51 | I can’t tell if cars passing me on the street are going at different speeds. | Hyposensitivity | Vision |
| 52 | I would be able to notice if someone added 5 grains of sugar to my glass of water. | Hypersensitivity | Taste |
| 53 | I would have difficulty seeing a single leaf clearly even on a tree that is close up. | Hyposensitivity | Vision |
| 54 | I wouldn’t taste if someone added a whole teaspoon of salt to my glass of water. | Hyposensitivity | Taste |
| 55 | I would be able to feel the elastic holding up my socks if I stop and thought about it. | Hypersensitivity | Touch |
| 56 | I can’t taste the difference between ripe and non-ripe fruit. | Hyposensitivity | Taste |
| 57 | I would be able to stand on one foot for fifteen seconds without wobbling. | Excluded | Hearing |
| 58 | I would be able to taste the difference between apparently identical pieces of candy. | Hypersensitivity | Taste |
| 59 | I notice the weight and pressure of a hat on my head. | *Hyposensitivity* | Touch |
| 60 | I would feel if a single hair touched the back of my hand. | *Hyposensitivity* | Touch |
| 61 | If I was walking along, I would be able to feel a passing truck’s vibrations even if my eyes were closed. | Excluded | Touch |
| 62 | I would be able to smell the smallest gas leak from anywhere in the house. | Hypersensitivity | Smell |
| 63 | I wouldn’t notice if someone changed their perfume, by smell alone. | Hyposensitivity | Smell |
| 64 | I would be able to tell when an elevator/lift started moving. | *Hyposensitivity* | Hearing |
| 65 | I can hear dog whistles very easily in the park. | Hypersensitivity | Hearing |
| 66 | I wouldn’t taste the difference between different types of lettuce leaves. | Hyposensitivity | Taste |
| 67 | I couldn’t taste if there were two slices of lemon in my glass of water if I was drinking it with my eyes closed. | Hyposensitivity | Taste |
| 68 | I can’t go out in bright sunlight without sunglasses. | Hypersensitivity | Vision |
| 69 | I would be able to read small print, such as a serial number on the back of a DVD, at 10 feet (3 metres) away. | Hypersensitivity | Vision |
| 70 | I get motion sickness easily (e.g., car sickness or sea sickness). | Hypersensitivity | Vision |
| 71 | I would be able to feel a change in the temperature of a cup of coffee after it had sat for 1 minute. | Hypersensitivity | Touch |
| 72 | I can’t hear very low frequency sounds, such as low voices. | Hyposensitivity | Hearing |
| 73 | I would be the first to hear if there was a fly in the room. | Hypersensitivity | Hearing |
| 74 | If I look at a pile of blue sweaters in a shop that are meant to be identical, I would be able to see differences between them. | Hypersensitivity | Vision |
| 75 | I wouldn’t detect a new smell in my house instantly before anyone else. | Hyposensitivity | Smell |
| 76 | I have perfect pitch: e.g. I could repeat a musical tone without any cue. | Hypersensitivity | Hearing |
| 77 | I would be able to bite into a lemon without any problems. | Hyposensitivity | Taste |
| 78 | I wouldn’t need to wear a coat in the winter, even when it is zero degrees outside. | Hyposensitivity | Touch |
| 79 | I wouldn’t be able to match the colour of a sweater in the shop with the colour of my trousers at home. | Excluded | Vision |
| 80 | I wouldn’t hear every single note when listening to music. | Excluded | Hearing |
| 81 | I would be able to smell the difference between most men and women. | Excluded | Smell |
| 82 | I choose to wear muted colours. | Excluded | Vision |
| 83 | I listen to music at minimum loudness. | Hypersensitivity | Hearing |
| 84 | I would be able to hear each note in a chord even if there were 10 notes. | Hypersensitivity | Hearing |
| 85 | I close curtains to avoid bright lights. | Hypersensitivity | Vision |
| 86 | I wouldn’t be able to hear differences in sound if the same instrument played the same note at different times. | *Hyposensitivity* | Hearing |
| 87 | I would be able to distinguish two brands of coffee by their smell, even with my eyes closed. | Hypersensitivity | Smell |
| 88 | I can see dust particles in the air in most environments. | Hypersensitivity | Vision |
| 89 | I wouldn’t be able to taste the difference between two brands of tomato sauce if they had different concentrations of salt. | Hyposensitivity | Taste |
| 90 | I would be able to smell the smallest amount of burning from anywhere in the house. | Hypersensitivity | Smell |
| 91 | If my mobile phone was vibrating in my pocket I would be quick to sense it. | *Hyposensitivity* | Touch |
| 92 | I find it difficult to see individual stars on a clear night | Hyposensitivity | Vision |
